# High-Fat Diet-Induced Obese Effects of Adipocyte-Specific CXCR2 Conditional Knockout in the Peritoneal Tumor Microenvironment of Ovarian Cancer

**DOI:** 10.3390/cancers13195033

**Published:** 2021-10-08

**Authors:** Deokyeong Choe, Eun-Sook Lee, Alicia Beeghly-Fadiel, Andrew J. Wilson, Margaret M. Whalen, Samuel E. Adunyah, Deok-Soo Son

**Affiliations:** 1 School of Food Science and Biotechnology, Kyungpook National University, Daegu 41566, Korea; cd02da@knu.ac.kr; 2 Department of Pharmaceutical Sciences, College of Pharmacy, Florida A&M University, Tallahassee, FL 32301, USA; eunsook.lee@famu.edu; 3 Division of Epidemiology, Department of Medicine, Vanderbilt University Medical Center, Nashville, TN 37203, USA; alicia.beeghly@vumc.org; 4 Vanderbilt-Ingram Cancer Center, Vanderbilt University Medical Center, Nashville, TN 37203, USA; andrew.j.wilson@vumc.org; 5 Department of Obstetrics and Gynecology, Vanderbilt University Medical Center, Nashville, TN 37232, USA; 6 Department of Chemistry, Tennessee State University, Nashville, TN 37209, USA; mwhalen@tnstate.edu; 7 Department of Biochemistry, Cancer Biology, Neuroscience and Pharmacology, School of Medicine, Meharry Medical College, Nashville, TN 37208, USA; sadunyah@mmc.edu

**Keywords:** obesity, high-fat diet, CXCR2, ascites, ovarian cancer

## Abstract

**Simple Summary:**

Obesity contributes to one-fifth of cancer deaths. Ovarian cancer (OC) progression is frequently asymptomatic, making its early detection difficult and its chance of survival low. OC expresses highly tumorigenic chemokines, CXCL1/8, of which the specific receptor CXCR2 is increased in the adipocytes. So, the CXCL1/8-CXCR2 axis may appear as a molecular link between obesity and OC. Here, we generated adipocyte-specific CXCR2 conditional knockout (cKO) mice to investigate how this CXCR2 cKO affects the peritoneal dissemination of OC under obese conditions. High-fat, diet-induced obese mice had a shorter survival than lean mice. Particularly, obese cKO mice had a reduced tumor burden but increased ascites accumulation, showing a decreased floating tumor burden in ascites, as well as proliferation and macrophage infiltration in tumors compared to obese wild-type mice. Despite the ascites accumulation, adipocyte-specific CXCR2 cKO reduced the obesity-induced tumor burden, likely altering the peritoneal tumor microenvironment of OC.

**Abstract:**

Obesity contributes to ovarian cancer (OC) progression via tumorigenic chemokines. Adipocytes and OC cells highly express CXCR2, and its ligands CXCL1/8, respectively, indicating that the CXCL1/8-CXCR2 axis is a molecular link between obesity and OC. Here, we investigated how the adipocyte-specific CXCR2 conditional knockout (cKO) affected the peritoneal tumor microenvironment of OC in a high-fat diet (HFD)-induced obese mouse model. We first generated adipocyte-specific CXCR2 cKO in mice: adipose tissues were not different in crown-like structures and adipocyte size between the wild-type (WT) and cKO mice but expressed lower levels of CCL2/6 compared to the obese WT mice. HFD-induced obese mice had a shorter survival time than lean mice. Particularly, obese WT and cKO mice developed higher tumors and ascites burdens, respectively. The ascites from the obese cKO mice showed increased vacuole clumps but decreased the floating tumor burden, tumor-attached macrophages, triglyceride, free fatty acid, CCL2, and TNF levels compared to obese WT mice. A tumor analysis revealed that obese cKO mice attenuated inflammatory areas, PCNA, and F4/80 compared to obese WT mice, indicating a reduced tumor burden, and there were positive relationships between the ascites and tumor parameters. Taken together, the adipocyte-specific CXCR2 cKO was associated with obesity-induced ascites despite a reduced tumor burden, likely altering the peritoneal tumor microenvironment of OC.

## 1. Introduction

Obesity, a disease defined by excessive fat accumulation which impairs health, is an escalating global epidemic: according to the World Health Organization, there are predicted to be 1.12 billion obese adults by 2030 [1]. Obesity is a known risk factor for many diseases, such as diabetes, cardiovascular disease, and cancer [2]. Several types of cancers are related to obesity, including cancers of the pancreas, corpus uteri (endometrium), esophagus, rectum, kidney, liver, colon, gallbladder, breast, thyroid, and ovary [3]. Obesity contributes to one-fifth of cancer deaths, with abnormal metabolism, chronic inflammation, growth factor signaling, and angiogenesis being the primary drivers of obesity-related cancers [4,5]. The direct molecular factors linking obesity and cancer are not fully understood, but the organ-dependent crosstalk between the adipose tissue and carcinomas via the vascular endothelial growth factor (VEGF), interleukin 6 (IL-6), tumor necrosis factor α (TNFα), and other mechanisms are implicated [6].

Among obesity-related cancers, ovarian cancer (OC) is the second leading cause of gynecological cancer-related death in women worldwide [7]. The asymptomatic and rapid progression of OC makes its early detection difficult, resulting in a low 5-year survival rate of only 49.1% [8]. This low survival rate is also attributable to the frequent and aggressive metastasis of OC throughout the peritoneal cavity, forming the secondary tumor foci and ascites [9]. The accumulation of malignant ascites provides growth factors, fatty acids, and chemokines to the OC cells, leading to a more aggressive and metastatic OC and a poor prognosis [10,11]. Chemokines are recognized as critical mediators in the tumor microenvironment and play a central role in regulating the inflammatory response, contributing to the progression and metastasis of OC [12]. Specifically, chemokines, such as CCL20, CXCL1–3, CXCL5, and CXCL8, are highly expressed in OC cells and the tumor microenvironment of OC [13,14,15]. These proangiogenic and tumorigenic chemokines are closely associated with the CXC chemokine receptor type 2 (CXCR2) [16,17,18,19]. CXCR2 is highly expressed in tumors of some OC patients and may promote OC progression [15,17]. In addition, CXCR2-mediated signaling is involved in the tumorigenicity of OC [18,19]. Interestingly, mouse adipocytes expressed higher levels of CXCR2 mRNA compared to preadipocytes [20] and OC progression could be accelerated by an obesity-induced inflammatory burden [21]. Based on the high expression levels of CXCL1–3 and CXCL8 in OC cells [13,14] and the high levels of CXCR2 in adipocytes [20], the CXCL1/8-CXCR2 axis is proposed as a molecular link between obesity and OC. CXCR2 transfected OC cells expressed higher levels of CXCL1/2 compared to CXCR2 null OC cells [19] and obesity-induced, higher circulating CXCL1 levels [21], indicating a positive feedback loop. CXCR2 knockout (KO) mice had a significantly reduced tumor burden in other cancers, such as murine lung, prostate, and renal cancer models, compared to CXCR2 wild-type (WT) mice [22,23,24]. These findings indicate that CXCR2 may be a critical molecular target to attenuate the progression of OC. However, the global deletion of CXCR2 using KO mice is not suitable for a diet-induced obesity model due to fewer and smaller adipocytes in this KO mouse model [25]. Accordingly, we generated adipocyte-specific, CXCR2 conditional knockout (cKO) mice for the first time to minimize the undesirable effects of CXCR2 deletion in other tissues, including immune cells. These CXCR2 cKO mice were employed to clarify the roles of adipocyte-derived CXCR2 signaling in the peritoneal dissemination of OC.

In this study, we investigated if adipocyte-specific CXCR2 cKO could modulate the peritoneal dissemination of OC in a pseudo-postmenopausal, diet-induced obese mouse model. We assessed the peritoneal tumor burden of OC under lean and obese states by feeding a normal diet (ND) and a high-fat diet (HFD), respectively, in adipocyte-specific CXCR2 cKO and WT mice.

## 2. Materials and Methods

### 2.1. Generation of Adipocyte-Specific CXCR2 cKO Mice

**Figure 1:**
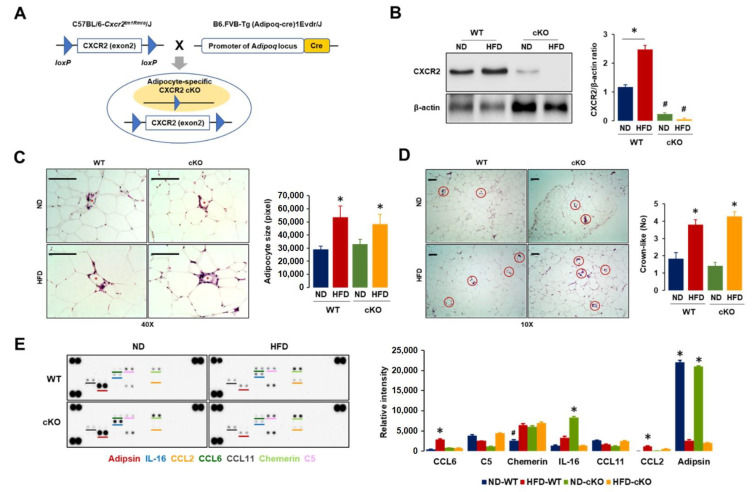
Generation of adipocyte-specific CXCR2 cKO mice and characteristics of adipose tissues in ND-WT, ND-cKO, HFD-WT, and HFD-cKO mice. (**A**) Generation of adipocyte-specific CXCR2 cKO mice by crossing C57BL/6-*Cxcr2^tm1Rmra^*/J (JAX #024638) with B6.FVB-Tg (Adipoq-cre)1Evdr/J (JAX #028020). (**B**) Confirmation of CXCR2 protein levels in perigonadal adipose tissues from ND-WT, ND-cKO, HFD-WT, and HFD-cKO mice without tumors. The image shown is representative of triplicate experiments. (**C**) Comparison of adipocyte size in perigonadal adipose tissues from ND-WT, ND-cKO, HFD-WT, and HFD-cKO mice. Red asterisks indicate crown-like structures and area measurement was calculated from 100 random adipocytes with ImageJ. (**D**) Comparison of crown-like structures in perigonadal adipose tissues from ND-WT, ND-cKO, HFD-WT, and HFD-cKO mice. Red circles mark crown-like structures; data were obtained from 10 random fields under microscope. (**E**) Comparison of chemokine/cytokine signature in perigonadal adipose tissues from ND-WT, ND-cKO, HFD-WT, and HFD-cKO mice by Proteome Profiler Mouse Chemokine Array (*n* = 3). Bars and error bars refer to mean and SEM. * and # indicates a significant increase and decrease (*p* ≤ 0.05), respectively, as determined by ANOVA and Tukey’s pairwise comparison.

We generated adipocyte-specific CXCR2 cKO mice with cre-lox technology by crossing C57BL/6-*Cxcr2^tm1Rmra^*/J (JAX #024638) with B6.FVB-Tg (Adipoq-cre)1Evdr/J (JAX #028020) under the cooperation of Jackson Laboratory (Bar Harbor, ME, USA). Homozygous (CXCR2fl) females were used as WT counterpart mice (Figure 1A).

### 2.2. Western Blots

Adipose tissues were excised from ND-WT, ND-cKO, HFD-WT, and HFD-cKO mice, and homogenized in a radio-immunoprecipitation assay buffer with protease and phosphatase inhibitors (MilliporeSigma, St. Louis, MO, USA). The homogenized mixtures were centrifuged at 10,000× *g* for 10 min at 4 °C and each clear supernatant, excluding the upper layer of lipid, was transferred to a new tube. After additional centrifugation under the same conditions, protein lysates were prepared. Total protein concentration for protein lysates was measured using the Bio-Rad protein assay based on the Bradford dye-binding method (Bio-Rad, Hercules, CA, USA). The protein lysates were fractionated on SDS-polyacrylamide gels and transferred to polyvinylidene difluoride membranes according to established procedures [19]. IL-8RB (K-19) antibody (Santa Cruz Biotechnology, Santa Cruz, CA, USA) was used to detect CXCR2 protein levels. β-actin (C4) antibody (Santa Cruz Biotechnology) served as an internal loading control. The targeted protein bands were visualized by chemiluminescence detection kits (MilliporeSigma, St. Louis, MO, USA).

### 2.3. Histological Analysis of Adipose Tissues

Perigonadal adipose samples from CXCR2 WT and cKO mice fed with ND and HFD without tumors were fixed in neutral buffered formalin for 7 days and then processed for paraffin embedding. Samples were sectioned at 5 μm and then processed for hematoxylin and eosin (H&E) staining. Digital images of slides were captured with BZ-X700 All-in-One Fluorescence Microscope (KEYENCE, Itasca, IL, USA). Adipocyte size was calculated as the mean area of 100 random adipocytes in digital images with ImageJ. Crown-like structures were counted from 10 random fields in microscopic images with 10× power fields.

### 2.4. Proteomic Array for Chemokine Signature

Chemokine signatures in adipose tissues and ascites were evaluated using Proteome Profiler Mouse Chemokine Array (ARY020; R&D Systems, Minneapolis, MN, USA) according to the manufacturer’s instructions as described previously [21]. Spot intensity of spots was calculated with ImageJ by subtracting average background signals and normalizing with reference spots.

### 2.5. Mouse Peritoneal Dissemination of OC in a Postmenopausal Obese Mouse Model

Mouse peritoneal dissemination of OC and diet-induced obesity at a pseudo-postmenopausal state were performed under guidelines approved by the Institutional Animal Care and Use Committee at Meharry Medical College (eProtocol#16-06-566) and the National Institutes of Health (NIH) guide for the Care and Use of Laboratory Animals. Six- to eight-week-old CXCR2 WT and cKO mice were ovariectomized (OVX) to create a pseudo-postmenopausal state. The mice were maintained in a specific pathogen-free animal housing facility at 22 °C ± 2 °C and 40–60% humidity under a 12:12 light: dark cycle. The mice were maintained on ND (5% kcal from fat) and HFD (D12492; 60% kcal from fat) obtained from Research Diets Inc. (New Brunswick, NJ, USA) for 16 weeks to produce lean and obese mice, respectively. We grouped mice as follows: (1) ND-WT; (2) HFD-WT; (3) ND-cKO; and (4) HFD-cKO. After confirming body weight gain in obese mice, mouse ID8 OC cells (3 × 10^6^ cells/mouse in a volume of 0.2 mL PBS) were injected intraperitoneally into all mice. Along with weekly body weight, mice were monitored 3 times per week to assess animal health such as hunched posture, lethargy and inactivity, impaired ambulation, shallow or labored breathing, hair coat condition, and change in the body weight. Mice showing signs of increase in body weight due to tumors and changes in appearance and activity were observed daily. When an increase of 20% in body weight, extensive ascites accumulation, or sluggish activity were observed, animals were euthanized for humane reasons. Tumors in the diaphragm, omentum, and pelvic sites were investigated for spreading index and omental tumor tissues were served for histological examination using H&E staining and immunohistochemistry with targeted antibodies. The survival times of the mice were compared between each group.

### 2.6. Cellular Characteristics in OC-Induced Ascites

Ascites collected via intraperitoneal aspiration were immediately smeared on slides and the slides were fixed in methanol and stained with Wright’s Giemsa stain. Ascitic fluid was prepared by centrifuging at 1500 rpm for 5 min and stored in a −80 °C freezer for further analysis. Digital images of slides were captured with BZ-X700 All-in-One Fluorescence Microscope (KEYENCE, Itasca, IL, USA). Floating cells in OC-induced ascites were quantitatively analyzed by calculating from 5 random fields of each slide using ImageJ for area measurement. Numbers of monocytes (Mo) and macrophages (Mφ) in OC-induced ascites were obtained from 5 random fields of each slide under microscope. Comparisons of vacuole and non-vacuole clumps showing cytoplasmic lipid droplets were obtained from 10 random fields of each slide under microscope.

### 2.7. Biochemical Analyses in OC-Induced Ascites

Triglyceride and free fatty acid levels in ascites were measured by EnzyChrom™ Triglyceride (ETGA-200) and Free Fatty Acid (EFFA-100) Assay Kits (BioAssay Systems, Hayward, CA, USA) according to the manufacturer’s instructions. The optical density of each well was determined using a microplate reader at 570 nm wavelength. Total protein levels for ascites were measured using the Bio-Rad protein assay based on the Bradford dye-binding method (Bio-Rad). Glucose levels in ascites were measured by Glucose Colorimetric Assay Kit (Cayman Chemical, Ann Arbor, MI, USA). The absorbance of each well was determined using a microplate reader at 514 nm wavelength. Vascular endothelial growth factor (VEGF) levels in ascites were measured by Mouse VEGF Quantikine ELISA Kit (MMV00; R&D Systems, Minneapolis, MN, USA) according to the manufacturer’s instructions. The optical density of each well was determined using a microplate reader set to 450 nm with wavelength correction of 570 nm. Tumor necrosis factor-α (TNFα) levels in ascites were measured by Mouse TNFα enzyme-linked immunosorbent assay (ELISA) kit (RAB0477; MilliporeSigma) according to the manufacturer’s instructions. The optical density of each well was determined using a microplate reader at 450 nm wavelength.

### 2.8. Histological and Immunohistochemical Evaluation of Tumor Tissues

Omental tumor samples were analyzed using H&E stain and immunohistochemistry. Paraffin slide sections were washed three times in xylenes followed by rehydration in a series of two alcohol washes (100% and 95%). Sections were treated for heat antigen retrieval in EDTA solution (1 mM, pH 8.0). After blocked in goat serum, sections were incubated overnight with specific primary antibodies as follows: PCNA (PC10) for proliferating cells, F4/80 (D2S9R) for Mφ, Arginase-1 (D4E3M) for MDSCs, CD4 (D7D2Z) for CD4 T cells, and CD8α (D4W2Z) for CD8 T cells (Cell Signaling Tech., Danvers, MA, USA). Sections were incubated with secondary antibody matched with primary antibody and developed using SignalStain^®^ DAB Substrate kit (#8059; Cell Signaling Tech.) with hematoxylin counterstain. Digital images of slides were captured with BZ-X700 All-in-One Fluorescence Microscope (KEYENCE). Quantitative analysis of inflammatory area in H&E stain was calculated using ImageJ with selection of inflammatory area through color threshold followed by particles analysis. Quantitative analysis of PCNA and immune cell dispositions was performed using ImageJ with brown color selection through color deconvolution of hematoxylin and 3,3′-diaminobenzidine (DAB) staining followed by intensity analysis. Quantitative analysis was obtained from 3–5 random fields of each slide (10×).

### 2.9. Statistical Analysis

Data values were expressed as the mean ± the standard error of the mean (SEM). Data were analyzed and compared using unpaired Student’s t-tests and one-way ANOVA as appropriate. If statistical significance (*p* ≤ 0.05) was determined by ANOVA, the data were further analyzed by Tukey’s pairwise comparison to detect specific differences between treatment groups. R-squared values for correlations between continuous variables were calculated from linear regression from the Data Analysis Tools in MS-Excel. Differences in survival plots were evaluated with the log-rank test [26].

## 3. Results

### 3.1. Adipocyte-Specific CXCR2 cKO Mice Have Low Levels of CCL2/6 in Adipose Tissues Compared to Those in CXCR2 WT Mice

CXCR2-mediated signaling was involved in the tumorigenicity of OC [18,19] and adipogenesis [20]. An adipocyte-driven chemokine network appeared to involve CXCR2-mediated signaling [20,25,27], launching a tumorigenic burden of OC cells which expressed high levels of CXCL1–3 and CXCL8 [13,14]. Accordingly, targeting CXCR2 may be critical in blocking the obesity-induced progression of OC. CXCR2 KO mice (C129S2(B6)-*Cxcr2^tm1Mwm^*/J mice, Jackson Laboratory) were not susceptible to diet-induced obesity, showing a lower weight gain. Thus, we first generated adipocyte-specific CXCR2 cKO mice by crossing C57BL/6-*Cxcr2^tm1Rmra^*/J (JAX #024638) with B6.FVB-Tg (Adipoq-cre)1Evdr/J (JAX #028020) (Figure 1A). All mice were ovariectomized to avoid the suppressive effects of estrogen on obesity [28,29] and to create a pseudo-postmenopausal state, as most ovarian cancers develop after menopause. Before the intraperitoneal injection of ID8 OC cells, we confirmed the predominant expression levels of the CXCR2 protein in the adipose tissues from ND- and HFD-fed WT mice, but little expression of CXCR2 in ND- and HFD-fed cKO mice (Figure 1B and Figure S1). The adipose tissues in HFD-fed WT mice showed a larger increase in CXCR2 protein levels compared to ND-fed mice (Figure 1B), indicating an obesity-induced CXCR2 expression. The adipocyte size showed an approximate 2-fold increase in HFD-fed mice compared to ND-fed mice but there was no significant difference between CXCR2 WT and cKO mice (Figure 1C). Crown-like structures in the adipose tissues were not different between CXCR2 WT and cKO mice but there was an approximate 2-fold increase in both HFD-fed mice (Figure 1D), indicating the stimulation of the proinflammatory processes in the adipose tissue due to obesity in HFD-fed mice. The chemokine signatures in adipose tissues revealed the induction of high levels of CCL2 and CCL6 proteins in HFD-fed WT mice, but not in HFD-fed cKO mice (Figure 1E and Figure S2). Interestingly, ND-fed WT and cKO mice showed low levels of chemerin and high levels of IL-16 in the adipose tissues, respectively (Figure 1E). HFD-fed mice had low levels of adipsin compared to ND-fed mice (Figure 1E). A decreased adipsin activity was observed as a common feature of several experimental models of obesity [30], validating the burden of obesity induced by HFD.

### 3.2. Obese CXCR2 WT Mice Have Greater Tumor Burdens, While Obese CXCR2 cKO Mice Have Greater Ascites Burdens

**Figure 2:**
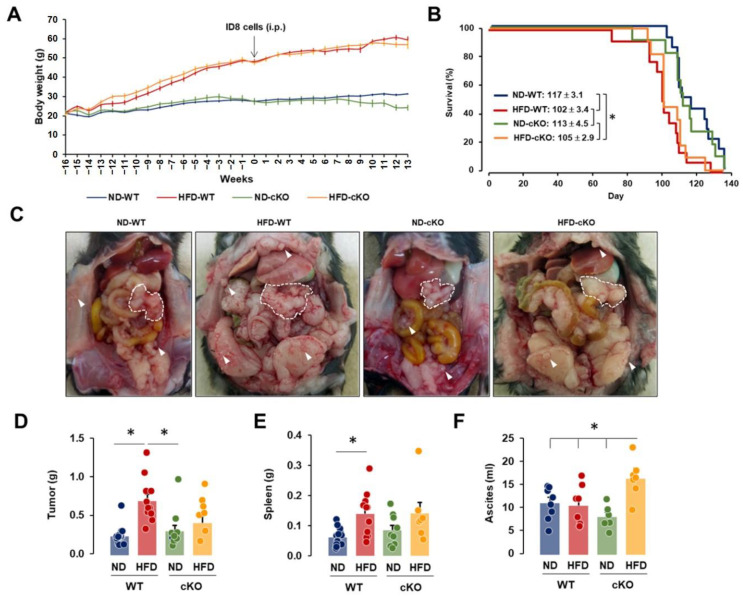
The peritoneal dissemination profiles of OC in ND-WT, ND-cKO, HFD-WT, and HFD-cKO mice. (**A**) Body weight trends in lean and obese CXCR2 WT and cKO mice treated intraperitoneally (i.p.) with mouse ID8 OC cells. Ovariectomized lean and obese mice were fed with ND and HFD throughout the whole experimental period, respectively. ND-WT (*n* = 14), ND-cKO (*n* = 11), HFD-WT (*n* = 15), and HFD-cKO (*n* = 12). The results were summed from 5 independent experiments because of different production numbers of CXCR2 cKO mice. (**B**) Overall survival rate in ND-WT, ND-cKO, HFD-WT, and HFD-cKO mice. * indicates significant difference (*p* ≤ 0.05) as calculated by the log-rank test. (**C**) Tumor burden and omental tumor tissues in the peritoneal cavity of ID8 OC cells bearing mice. Bold white dots and arrows indicate the omental OC burden and tumor spread in the parietal and visceral peritoneum, respectively. Representative pictures were obtained from each group. Comparison of (**D**) tumor weight, (**E**) spleen weight, and (**F**) ascites volumes in ND-WT (*n* = 8 for ascites and 14 for tumor and spleen), ND-cKO (*n* = 6 for ascites, 10 for tumor, and 9 for spleen), HFD-WT (*n* = 8 for ascites and 11 for tumor and spleen), and HFD-cKO (*n* = 7 for all parameters) mice. * indicates a significant difference (*p* ≤ 0.05) between groups as analyzed by ANOVA and Tukey’s pairwise comparison tests.

There were no significant differences in body weight between WT and cKO mice at the start of our experiments and in the body weight gain after ND and HFD conditions (Figure 2A). The HFD-fed obese mice had approximately 2-fold higher body weights compared to ND-fed lean mice. Although lean CXCR2 cKO mice had a decreasing trend in body weight with an advanced tumor burden (from 9 weeks after OC cell injection), this was not significant (Figure 2A). HFD-fed obese mice had a shorter survival than ND-fed mice, regardless of the CXCR2 cKO in the adipose tissues, with outcomes of 117 ± 3.1 days in ND-WT, 102 ± 3.4 days in HFD-WT, 113 ± 4.5 days in ND-cKO, and 105 ± 2.9 days in HFD-cKO mice (Figure 2B). Although our results supported a shorter survival in OC patients with obesity [31,32], adipocyte-specific CXCR2 cKO was not likely to critically affect the survival of OC. The peritoneal dissemination of ID8 OC cells was observed in all mice, spreading widely into the parietal and visceral peritoneum with a solid tumor formation on the omentum (Figure 2C). The obese WT mice had increased tumor weights compared to the lean mice, while obese cKO mice tended to have higher tumor weights without statistical significance (Figure 2D). The spleen weights shared a similar pattern (Figure 2E). Interestingly, obese cKO mice had a greater accumulation of ascites in the peritoneal cavity compared to other groups (Figure 2F). This indicated that adipocyte-specific CXCR2 cKO was related to the obesity-induced accumulation of ascites in OC.

### 3.3. Adipocyte-Specific CXCR2 cKO Mice Have Lower Floating Tumor Burdens and Tumor-Attached Monocytes/Macrophages but Increased Vacuole Clumps in OC-Induced Ascites after Diet-Induced Obesity

**Figure 3:**
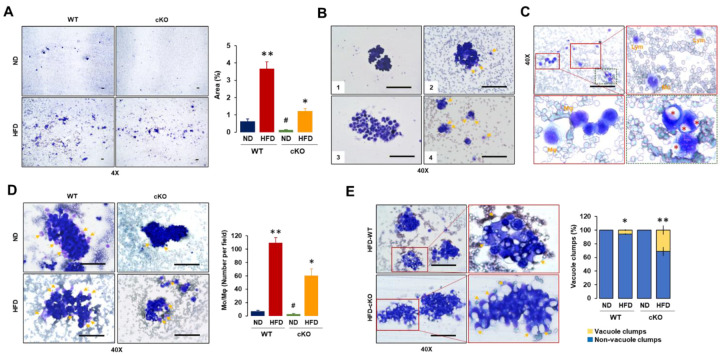
Cellular characteristics of OC-induced ascites in a postmenopausal, diet-induced, obese mouse model between CXCR2 WT and cKO mice. (**A**) Comparison of floating cells in OC-induced ascites obtained from lean and obese CXCR2 WT and cKO mice bearing mouse ID8 OC cells. Area measurement was calculated from 5 random fields of each slide with ImageJ. (**B**) Morphological patterns of floating tumor cells in OC-induced ascites. B1: intact floating tumor cells; B2: Mo/Mφ-attached floating tumor cells; B3: dispersed floating tumor cells with immune cells; B4: small clumps of floating tumor cells attached with monocyte/Mφ. Yellow arrows indicate Mo/Mφ. (**C**) Morphological patterns between immune and tumor cells in OC-induced ascites. Lym: lymphocytes. Red asterisks: signet ring-like cells containing cytoplasmic vacuoles. (**D**) Numerical comparison of Mo and Mφ in OC-induced ascites from ND-WT, ND-cKO, HFD-WT, and HFD-cKO mice. Yellow arrows indicate attached Mo and Mφ. Cell number was obtained from 5 random fields of each slide under microscope. (**E**) Comparison of vacuole and non-vacuole clumps in OC-induced ascites; clump number was obtained from 10 random fields of each slide under microscope. Yellow arrows indicate vacuoles. *, **, and # indicates significant difference (*p* ≤ 0.05) between groups as analyzed by ANOVA and Tukey’s pairwise comparison tests.

Because obese CXCR2 cKO mice had greater ascites burdens (Figure 2F), we performed ascitic analysis using ascites smeared with Wright’s Giemsa stain. The floating cells in the OC-induced ascites were categorized from large to small areas in the following order: HFD-WT > HFD-cKO > ND-WT > ND-cKO mice (Figure 3A). We observed the morphological patterns of floating tumor cells in OC-induced ascites as follows: intact floating tumor cells, Mo- and Mφ-attached floating tumor cells, dispersed floating tumor cells with immune cells, and small clumps of floating tumor cells attached with Mo/Mφ (Figure 3(B1–4)). Supposedly, the tumor cells floating without the attached immune cells seemed to be detached from the original tumor sites just before the immune cells attached to the tumor clumps (Figure 3(B1)). Immune cells, such as Mo and Mφ seemed to approach to tumor clumps in order to attack (Figure 3(B2)). Mo and Mφ attached to the tumor cells which seemed to disperse tumor clumps, impeding the tight attachment between tumor cells (Figure 3(B3)). Finally, the dispersed small tumor clumps seemed to be further attacked by immune cells, such as Mo and Mφ (Figure 3(B4)). We also observed some morphological patterns between immune and tumor cells in OC-induced ascites. Few lymphocytes and Mo were floating in the ascites (Figure 3C). Signet, ring-like cells containing cytoplasmic vacuoles were observed in HFD-fed mice but were rare in ND-fed mice (Figure 3C). The distribution of Mo and Mφ in OC-induced ascites descended in the following order: HFD-WT > HFD-cKO > ND-WT > ND-cKO mice (Figure 3D), being consistent with the floating tumor cells (Figure 3A). Almost all Mo and Mφ in the OC-induced ascites were attached to tumor clumps but small numbers of them were floating in the ascites. Vacuole clumps showing cytoplasmic lipid droplets in the OC-induced ascites were frequently observed in HFD-fed mice but very rarely in ND-fed mice, demonstrating their higher levels in obese CXCR2 cKO mice (Figure 3E).

### 3.4. Ascites in Obese CXCR2 cKO Mice Showed Lower Levels of Triglycerides, Free Fatty Acids, CCL2, and TNF Compared to Those in Obese WT Mice

**Figure 4:**
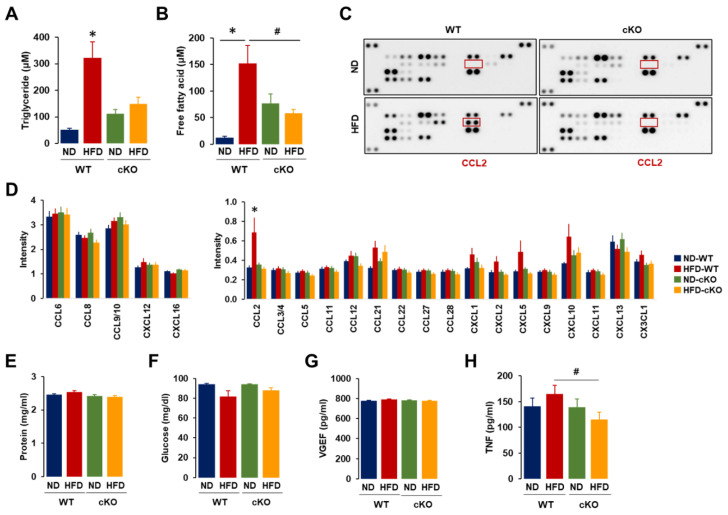
Biochemical characteristics of OC-induced ascites in a postmenopausal diet-induced obese mouse model between CXCR2 WT and cKO mice. (**A**) Triglyceride and (**B**) free fatty acid levels in OC-induced ascites of ND-WT (*n* = 8), ND-cKO (*n* = 6), HFD-WT (*n* = 8), and HFD-cKO (*n* = 7) mice using quantitative colorimetric assays with duplicate measurements. (**C**) Chemokine signatures in ascites of ND-WT, ND-cKO, HFD-WT, and HFD-cKO mice by proteomic array. (**D**) Intensity of chemokine expression profiles in OC-induced ascites of ND-WT, ND-cKO, HFD-WT, and HFD-cKO mice. (**E**) Total protein, (**F**) glucose, (**G**) VEGF, and (**H**) TNF levels in OC-induced ascites of ND-WT, ND-cKO, HFD-WT, and HFD-cKO mice. * and # indicates significant increases and decreases (*p* ≤ 0.05), respectively, between groups as analyzed by ANOVA and Tukey’s pairwise comparison tests.

The ascites from HFD-fed CXCR2 WT mice had high levels of triglycerides and free fatty acids (Figure 4A,B). Although the ascites from the ND-fed cKO mice showed high levels of triglycerides and free fatty acids compared to those from ND-fed WT mice, HFD was unable to significantly increase these levels in the OC-induced ascites of CXCR2 cKO mice (Figure 4A,B). Consequently, we evaluated if adipocyte-specific CXCR2 cKO influenced the chemokine signatures in OC-induced ascites using proteomic arrays. Although OC-induced ascites contained high levels of CCL6, CCL8, CCL9/10, CXCL12, and CXCL16, these chemokines showed no significant difference between obese and lean mice or between WT and cKO mice (Figure 4C,D and Figure S3). Interestingly, CCL2 protein levels were significantly higher in obese WT mice compared to those in all the other groups (Figure 4C,D). Although obese WT mice tended to show higher protein levels of CXCL1, CXCL2, CXCL5, and CXCL10 (Figure 4D), there were no statistical differences between groups. The total protein amount in ascites was similar between groups (Figure 4E), indicating that the reduced levels of CCL2 in the ascites of obese cKO mice were not due to differences in volume or dilution. The levels of glucose and VEGF in OC-induced ascites had no significant differences between the groups (Figure 4F,G), while obese cKO mice showed lower levels of secreted TNFα compared to those in obese WT mice (Figure 4H).

### 3.5. Obese CXCR2 cKO Mice Show Attenuated Inflammatory Area, PCNA, and F4/80 in Tumor Tissues Compared to Obese WT Mice

**Figure 5:**
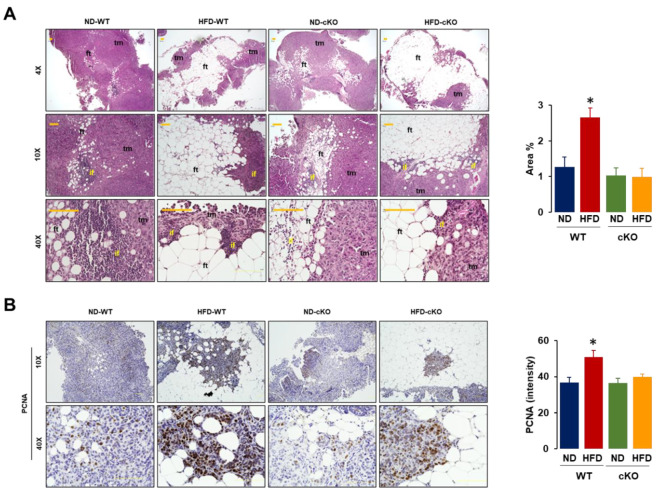
Histological evaluation of the omental tumor tissues in a postmenopausal diet-induced obese mouse model between CXCR2 WT and cKO mice. (**A**) Histological features of omental tumors in ND-WT, ND-cKO, HFD-WT, and HFD-cKO mice using H&E stain. tm: tumor tissue, ft: fat tissue, if: inflammatory region. Quantitative analysis of inflammatory area was performed using ImageJ with selection of inflammatory area through color threshold followed by particles analysis. (**B**) Disposition of PCNA positive cells in omental tumors from ND-WT, ND-cKO, HFD-WT, and HFD-cKO mice using immunohistochemistry. Quantitative analysis of PCNA disposition was performed using ImageJ with brown color selection through color deconvolution of hematoxylin and DAB staining followed by intensity analysis. Quantitative analysis was obtained from 3–5 random fields of each slide (10×). * indicates a significant difference (*p* ≤ 0.05) between groups as analyzed by ANOVA and Tukey’s pairwise comparison tests.

We evaluated the histological features of omental tumor tissues as a frequent site of OC in the peritoneal cavity. Both obese CXCR2 WT and cKO mice had larger and more abundant adipocytes in the omental tumor tissues compared to those in lean mice (Figure 5A). Particularly, the regions between the adipose and tumor tissues showed evidence of an inflammatory reaction as shown by the recruited immune cells; this was greater in HFD-fed WT mice compared to other groups (Figure 5A). Because proliferating the cell nuclear antigen (PCNA) was a universal marker of proliferating cells, we evaluated PCNA positive cells in omental tumor tissues. Most PCNA positive cells appeared at the edges of tumor tissues to rapidly develop tumor growth and regions between the adipose and tumor tissues (Figure 5B). A quantitative analysis of the PCNA disposition revealed that HFD-fed WT mice had greater PCNA-positive cells compared to other groups (Figure 5B).

**Figure 6:**
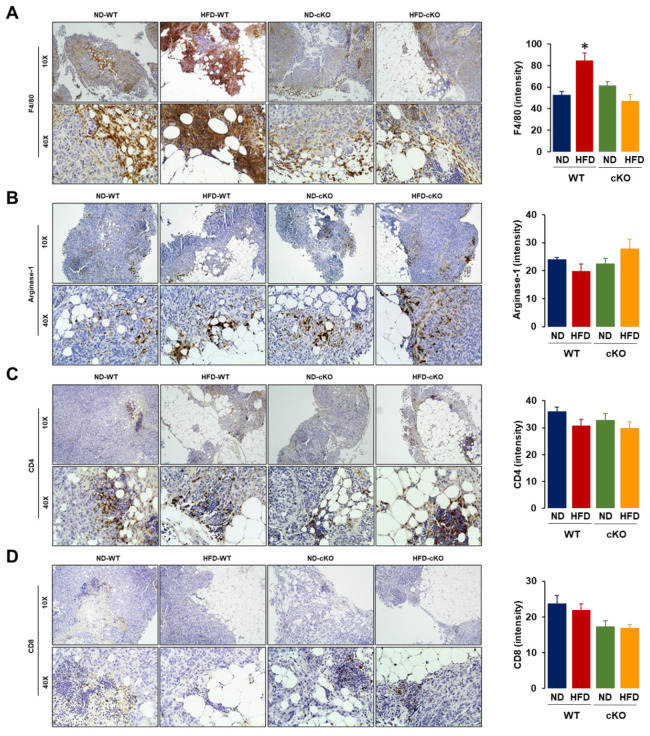
Immunohistochemical evaluation of immune cells in omental tumor tissues from a postmenopausal diet-induced obese mouse model between CXCR2 WT and cKO mice. Histological features of (**A**) F4/80-, (**B**) arginase-1-, (**C**) CD4-, and (**D**) CD8-positive cells in omental tumors from ND-WT, ND-cKO, HFD-WT, and HFD-cKO mice. Quantitative analysis of immune cell disposition was performed using ImageJ with brown color selection through color deconvolution of hematoxylin and DAB staining followed by intensity analysis. Quantitative analysis was obtained from 3–5 random fields of each slide (10×). * indicates a significant difference (*p* ≤ 0.05) between groups as analyzed by ANOVA and Tukey’s pairwise comparison tests.

Because tumor infiltrating immune cells were associated with clinical outcomes, we evaluated the immune cell infiltration in omental tumor tissues. F4/80, as a major Mφ marker, appeared intensively in regions between the fat and tumor tissues. A quantitative analysis of F4/80 disposition revealed that HFD-fed WT mice had greater F4/80-positive cells compared to the other groups (Figure 6A). The myeloid-derived suppressor cells (MDSCs) expressed high levels of arginase-1 and were recruited to the tumor tissues [33]. Most arginase-1 positive cells appeared at the edges of the tumor tissues to trigger tumor growth and in the regions between the adipose and tumor tissues. A quantitative analysis of arginase-1 disposition showed no significant difference between the groups (Figure 6B). Most CD4-positive cells appeared in the inflammatory regions between the adipose and tumor tissues, with no significant differences between the groups in the CD4 disposition (Figure 6C). Similar to the CD4 disposition, most of the CD8-positive cells also appeared in the inflammatory regions between the fat and tumor tissues, showing no significant difference between the groups in the CD8 disposition (Figure 6D).

### 3.6. CXCR2 cKO Mice Showed a Higher Positive Relationship between Ascites and Tumor Parameters Compared to WT Mice

**Figure 7:**
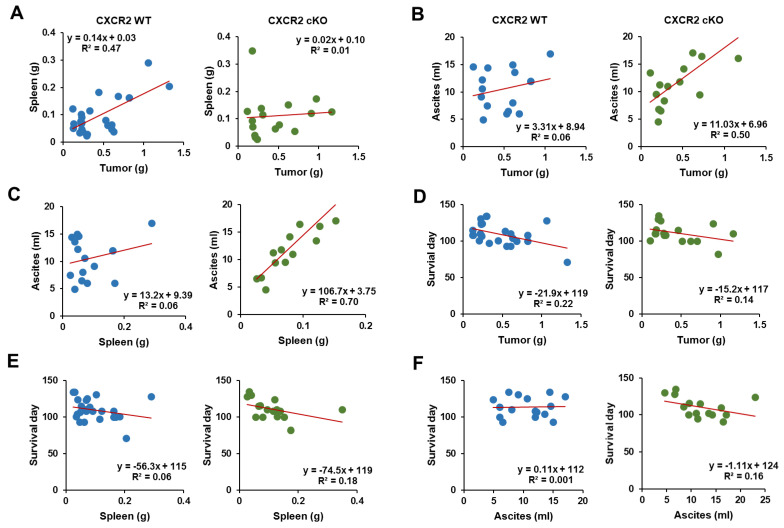
Correlations in tumor parameters between CXCR2 WT and cKO mice. (**A**) Correlation of tumor and spleen weights from CXCR2 WT and cKO mice. (**B**) Correlation of tumor weights and ascites volumes in CXCR2 WT and cKO mice. (**C**) Correlation of spleen weights and ascites volumes in CXCR2 WT and cKO mice. (**D**) Correlation of tumor weights and survival days in CXCR2 WT and cKO mice. (**E**) Correlation of spleen weights and survival days in CXCR2 WT and cKO mice. (**F**) Correlation of ascites volumes and survival days in CXCR2 WT and cKO mice.

Because obese CXCR2 WT and cKO mice had greater tumor weights and ascites accumulation (Figure 2D,F), respectively, we evaluated the correlations between the tumor weights, spleen weights, ascites accumulation, and survival between CXCR2 WT and cKO mice. CXCR2 WT mice had a higher correlation between the tumor and spleen weights (R^2^ = 0.47) than between the tumor weights and ascites (R^2^ = 0.06). On the contrary, the CXCR2 cKO mice had a higher correlation between the tumor weights and ascites (R^2^ = 0.50) than between the tumor and spleen weights (R^2^ = 0.01) (Figure 7A,B). Furthermore, CXCR2 cKO mice had a higher correlation between the spleen weights and ascites (R^2^ = 0.70) than WT mice (R^2^ = 0.06) (Figure 7C). With regard to survival, CXCR2 WT mice had a higher correlation with the tumor weights (R^2^ = 0.22) than with spleen weights (R^2^ = 0.06) and ascites (R^2^ = 0.001) (Figure 7D,F), whereas CXCR2 cKO mice had similar correlations with the tumor weights (R^2^ = 0.14), spleen weights (R^2^ = 0.18), and ascites (R^2^ = 0.16), demonstrating survival durations which lasted days (Figure 7D,F). These correlation results suggested that the tumor and ascites burdens may be critical for the survival of CXCR2 WT and cKO mice, respectively.

## 4. Discussion

Our main finding from the present study is that adipocyte-specific CXCR2 cKO resulted in a lower tumor burden but increased the OC-induced ascites under obesity-inducing diet conditions. On the contrary, CXCR2 WT mice seemed to have a higher tumor burden rather than an ascites burden from OC under obese conditions.

The crown-like structures in the adipose tissues indicated the adipose microenvironments of Mφ engulfing adipocytes, and their densities were usually higher in obese mice than in lean mice [34]. Because HFD-fed mice increased the crown-like structures compared to ND-fed mice without any change between CXCR2 WT and cKO mice (Figure 1C), adipocyte-specific CXCR2 cKO was unlikely to critically affect adipose tissue inflammation under obese conditions. The average size of adipocytes was larger in obese mice than in lean mice [35]. Consistent with the results, HFD-fed mice in our study showed an increased adipocyte size compared to that in ND-fed mice, but no difference between the CXCR2 WT and cKO mice (Figure 1D). Female CXCR2 KO mice had a thinner subcutaneous adipose layer due to fewer and smaller adipocytes, which was not observed in male mice [25]. In contrast to the systemic CXCR2 KO, the adipocyte-specific CXCR2 cKO may not critically affect adipose morphology. On the other hand, the obese CXCR2 cKO mice had low levels of CCL2/6 in the adipose tissues compared to obese WT mice (Figure 1E). The adipocytes produced CCL2 which was correlated with body mass index (BMI), whereas weight loss reduced CCL2 levels [36]. Surgery-induced weight loss also reduced urinary CCL2 levels [37]. Adipogenesis in mouse 3T3-L1 cells indicated CCL6 as a dominant chemokine in mouse adipocytes [20]. These reports supported the HFD-induced CCL2/6 levels in adipose tissues. Although CXCR2 cKO mice showed a normal adipose morphology, the adipocyte-specific CXCR2 cKO may have affected the adipose microenvironment by disrupting the adipose CCL2/6 levels in response to HFD. The CXCR2 cKO mice showed a similar weight trend with WT mice in response to ND and HFD (Figure 2A), supporting the similar results of crown-like structures and adipocyte size in adipose tissues between WT and cKO mice (Figure 1C,D).

As obesity increases the risk of OC progression and leads to enhanced tumor burden [38], HFD-induced obesity resulted in a shorter survival regardless of the CXCR2 in adipose tissues (Figure 2B). A shorter survival may be due to a greater tumor and ascites burden in the HFD-fed CXCR2 WT and cKO, respectively (Figure 2D,F). These results indicate that CXCR2-mediated signaling is involved in increased tumor burden in the tumor microenvironment of OC by interacting with adipose tissues. Although obese mice had a greater tumor burden compared to lean mice, the ascites accumulation was unchanged [21]. Because the increased OC-induced ascites in HFD-fed CXCR2 cKO mice (Figure 2F) was unexpected, we further analyzed the cellular and biochemical characteristics of ascites. Consistently, as ascites in *ob/ob* mice had higher levels of Mφ than lean mice [21], the HFD-fed CXCR2 WT mice demonstrated dominant floating tumor burdens and tumor-attached Mo/Mφ in OC-induced ascites. On the other hand, ascites in HFD-fed cKO mice attenuated the floating tumor burdens and tumor-attached Mo/Mφ compared to HFD-fed WT mice (Figure 3A,D). Even ND-fed cKO mice reduced these parameters in OC-induced ascites compared to ND-fed WT mice (Figure 3A,D). These results confirmed that the adipocyte-specific CXCR2 cKO attenuated the tumor burden of OC in response to HFD. CXCR2 KO mice also had a reduced tumor volume after the injection with breast cancer cells [39], supporting the attenuated tumor burdens in adipocyte-specific CXCR2 cKO mice. Interestingly, HFD-fed CXCR2 cKO mice showed increased cytoplasmic lipid droplets of cell clumps in the OC-induced ascites (Figure 3E). The relationship between the increased vacuole clumps and ascites volume was unclear at this point, requiring further study. The increased vacuole clumps may be associated with lower levels of triglycerides and free fatty acids as observed more in the OC-induced ascites of cKO mice than in WT mice (Figure 4A,B), forming further vacuole clumps to reduce the amount of fat in the ascites. On the other hand, the ascites of ND-fed cKO mice showed higher levels of triglycerides and free fatty acids compared to that of ND-fed WT mice (Figure 4A,B). These increased levels may be in part associated with decreased Mφ in ascites of ND-fed cKO mice (Figure 4D) because Mφ could accumulate lipids via phagocytosis. VEGF was a permeability factor for endothelial cells to play a role in angiogenesis [40]. Because VEGF levels in ascites was similar between WT and cKO mice (Figure 4G), VEGF was unlikely to be a factor for the OC-induced ascites in CXCR2 cKO mice. CXCR2-deficient mice had reduced the blood–brain barrier permeability in viral encephalitis [41] but the permeability was not affected by closed head injury [42], indicating a disease-specific response.

Among chemokine signatures in OC-induced ascites, CCL2 had a significantly higher expression in HFD-fed WT mice (Figure 4C,D), indicating that CCL2 may be an important player in the peritoneal microenvironment of OC under obese conditions. CCL2 is known to be markedly increased in obesity, with roles in Mo recruitment [43]. Our previous results also showed high levels of CCL2 in *ob/ob* mice with leptin deficiency [21]. On the other hand, HFD-fed CXCR2 cKO mice had lower levels of secreted TNFα compared to obese WT mice (Figure 4H). TNFα was shown to be significantly upregulated in ovarian tumor tissues, leading to NF-κB activation [44]. Because CCL2, CXCL1, CXCL2, CXCL5, and CXCL10 were upregulated by NF-κB signaling [14,45,46], TNFα-induced NF-κB activation may have contributed to the increased tendencies of these chemokines in HFD-fed WT mice (Figure 4D). Glioblastoma cell-secreted CXCL8 induced brain endothelial cell permeability via CXCR2 [47]. As the mice lacked IL8/CXCL8, the murine homologues for human CXCL8 were considered to be CXCL1–2 and CXCL5, as CXCR2 ligands [48]. Because CXCL1–2 and CXCL5 levels showed a decreased tendency in ascites of HFD-fed CXCR2 cKO mice, compared to the WT mice (Figure 4D), these chemokines were unlikely to contribute to OC-induced ascites in HFD-fed CXCR2 cKO mice. CCL2 secreted from cancer-associated mesothelial cells could promote the malignant potential of OC, playing a crucial role in the tumor microenvironment of OC [49]. However, the increased ovarian tumoral expression of CCL2 was associated with an improved response to chemotherapy and survival outcomes [50]. Despite the controversial effects of CCL2 in OC, the CCL2-CCR2 axis was recognized as a critical player in recruiting tumor-associated Mφ and facilitating the progression of cancer [51], indicating further promoting effects due to obesity. Although ID8 OC cells constitutively expressed CCL2 [46], adipocyte-conditioned media had no effects on CCL2 but instead induced CXCL1, CXCL2, and CXCL10 in ID8 cells [21]. Therefore, the higher levels of CCL2 in the ascites of obese WT mice may be derived not from tumoral tissues but from obesity-related, extra-tumoral tissues including adipocytes, as observed in adipose tissues (Figure 1E). Although showing a similar weight gain (Figure 2A) and similar distribution pattern of body fat (Figure 2C) with WT mice, obese CXCR2 cKO mice showed lower levels of triglyceride, FFA, CCL2, and Mφ in ascites compared to obese WT mice (Figure 4). These results indicated that CXCR2 cKO obesity critically responded to a pathological state rather than a physiological state. As a future research direction, the quantitative analysis of physiological aspects, such as the food intake pattern, fat distribution, and lipid metabolism, may give an important insight to clarify the underlying mechanisms affecting the roles of CXCR2 in obesity.

The tumor tissues in HFD-fed CXCR2 cKO mice had lower inflammatory areas and PCNA and F4/80 expressions, compared to those in HFD-fed WT mice (Figure 5 and Figure 6A), indicating a reduced tumor burden. These facts indicated that CXCR2-mediated signaling was involved in tumor burden, showing immune cell infiltration in the tumor microenvironment. Consistently, systemic CXCR2 KO mice decreased PCNA and F4/80 tumor associated Mφ in mammary tumors [39]. CXCR2 KO mice also suppressed acute and chronic pancreatic inflammation [52]. While CXCR2 KO mice decreased MDSCs in breast tumors [39], adipose-specific CXCR2 cKO mice had no change in omental tumor tissues of OC (Figure 6B). Particularly, the regions between the fat and tumor tissues were the main target areas for immune cell infiltration, indicating the immune cell-mediated interaction between the adipocytes and cancer cells followed by the higher potentiation of PCNA and F4/80-positive cells under obese conditions. In addition, based on the correlation results (Figure 7), CXCR2 WT and cKO mice could favorably induce tumor and ascites burdens, respectively, playing a critical role in OC survival.

**Figure 8:**
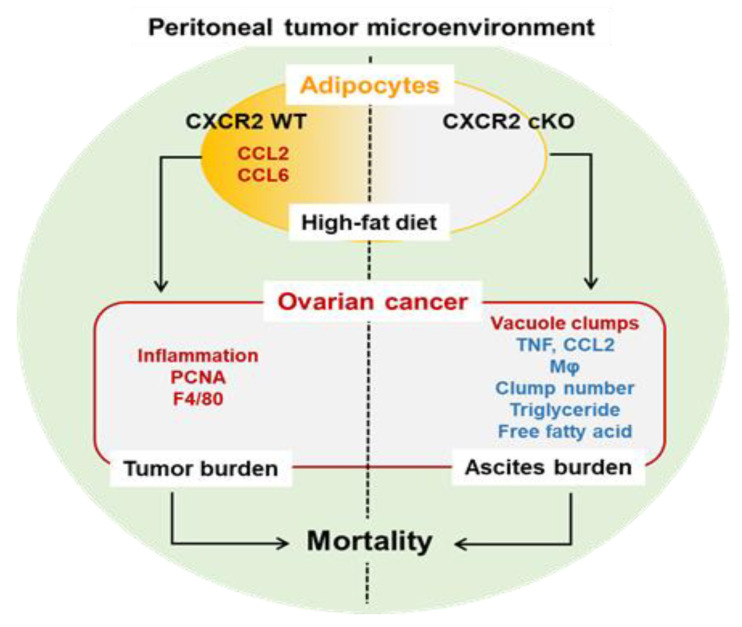
Schematic for HFD-induced obese effects of adipocyte-specific CXCR2 cKO on the peritoneal tumor microenvironment of OC. Adipose tissues express high levels of CCL2/6 in obese WT mice compared to those in obese cKO mice. Interestingly, obese CXCR2 WT and cKO mice appear to have greater tumor and ascites burdens, respectively. Compared to obese CXCR2 WT mice, the cKO mice had lower ascitic floating tumor burdens and tumor-attached Mo/Mφ, less triglyceride, free fatty acid, CCL2, and TNF levels, but increased vacuole clumps in OC-induced ascites. Obese CXCR2 WT mice had more inflammatory areas and PCNA- and F4/80-positive cells in tumor tissues compared to obese cKO mice. HFD-induced obesity leads to a shorter survival time compared to lean mice, regardless of CXCR2 status in adipose tissues. Therefore, adipocyte-specific CXCR2 cKO is associated with ascites accumulation under obese conditions, instead of tumor burden as shown in obese CXCR2 WT mice, most likely altering the peritoneal tumor microenvironment of OC. Red and blue letters indicate increases and decreases, respectively.

We summarized how adipocyte-specific CXCR2 cKO affected the peritoneal dissemination of OC under HFD-induced obese models based on our current results (Figure 8). As CCL2 is known to function in Mo/Mφ recruitment [43], the high levels of CCL2 in the adipose tissues and OC-induced ascites of HFD-fed WT mice could contribute to increased Mo/Mφ in the ascites and omental tumors. Overall, obese CXCR2 WT mice were likely prone to drive OC progression through tumor growth, while obese cKO mice favored ascites accumulation, which required further study to identify the molecular mechanisms.

## 5. Conclusions

In this study, we investigated how adipocyte-specific CXCR2 cKO affects the peritoneal tumor microenvironment of OC in an HFD-induced obese mouse model. Adipocyte-specific CXCR2 cKO mice fed with HFD had lower tumor burdens, inflammatory areas, proliferation markers, and Mφ infiltration compared to HFD-fed WT mice. However, obese CXCR2 cKO mice had a greater OC-induced ascites accumulation, showing reduced ascitic floating tumor cells and tumor-attached Mo/Mφ with lower triglyceride, free fatty acid, CCL2, and TNF levels compared to obese WT mice. Ascites in obese cKO mice had more vacuole clumps and a positive relationship with tumor and spleen weights. Therefore, adipocyte-specific CXCR2 WT and cKO were associated with tumor and ascites burdens, respectively, such that the loss of CXCR2 in adipocytes altered the peritoneal tumor microenvironment of OC.

## Data Availability

Not applicable.

## Supplementary Materials

The following are available online at https://www.mdpi.com/article/10.3390/cancers13195033/s1, Figure S1: The full western blot of Figure 1B, Figure S2: The full western blot of Figure 1E, Figure S3: The full western blot of Figure 4C.
