# Supplementary material for: High-Fat Diet-Induced Obese Effects of Adipocyte-Specific CXCR2 Conditional Knockout in the Peritoneal Tumor Microenvironment of Ovarian Cancer
Source: Cancers (Basel). 2021 Oct 8;13(19):5033. doi: 10.3390/cancers13195033 (PMC8508092; doi:10.3390/cancers13195033)
Supplement: Supplementary file 1 [file cancers-13-05033-s001.zip › cancers-1386083-Supplementary material.pdf]

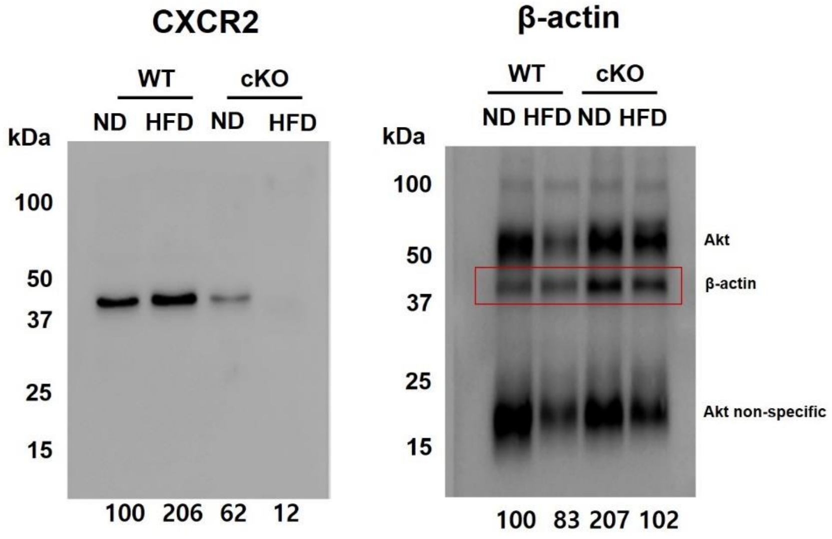

Figure S1. The full western blot of Figure 1B.

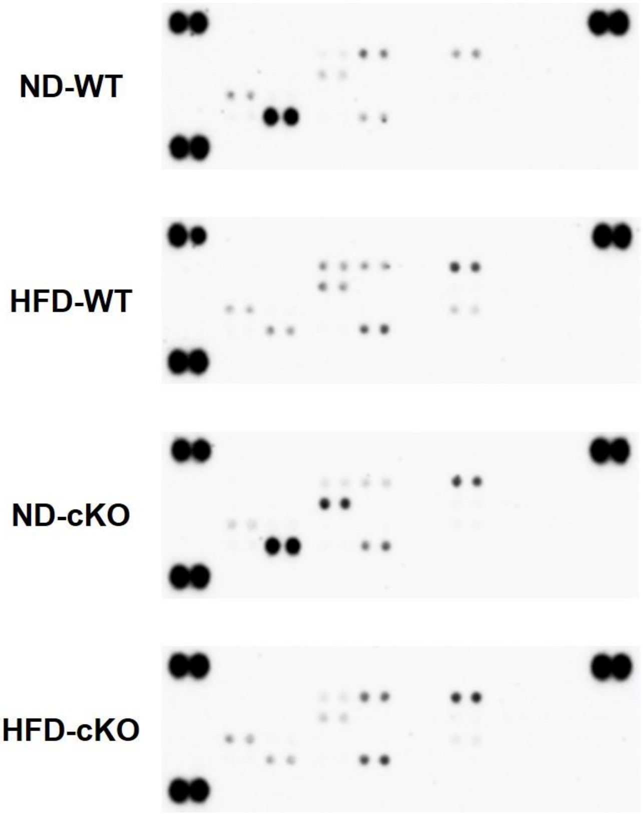

Figure S2. The full western blot of Figure 1E.

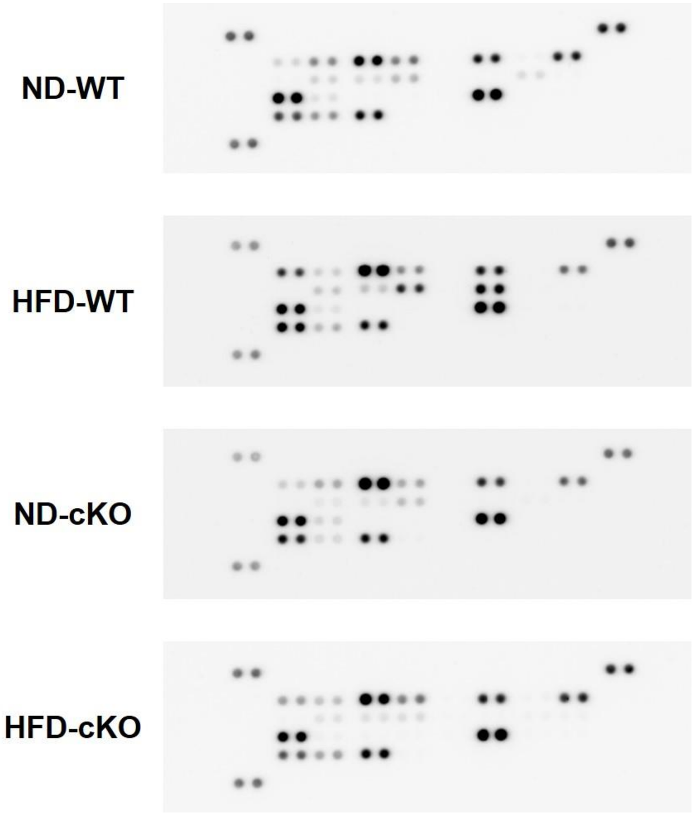

Figure S3. The full western blot of Figure 4C.
